# The Genetics of a Behavioral Speciation Phenotype in an Island System

**DOI:** 10.3390/genes9070346

**Published:** 2018-07-10

**Authors:** Thomas Blankers, Kevin P. Oh, Kerry L. Shaw

**Affiliations:** Department of Neurobiology and Behavior, Cornell University, Ithaca, NY 14853, USA; k.oh@colostate.edu (K.P.O.); kls4@cornell.edu (K.L.S.)

**Keywords:** speciation, sexual isolation, quantitative genetics, transcriptome, *Laupala*

## Abstract

Mating behavior divergence can make significant contributions to reproductive isolation and speciation in various biogeographic contexts. However, whether the genetic architecture underlying mating behavior divergence is related to the biogeographic history and the tempo and mode of speciation remains poorly understood. Here, we use quantitative trait locus (QTL) mapping to infer the number, distribution, and effect size of mating song rhythm variations in the crickets *Laupala eukolea* and *Laupala cerasina*, which occur on different islands (Maui and Hawaii). We then compare these results with a similar study of an independently evolving species pair that diverged within the same island. Finally, we annotate the *L. cerasina* transcriptome and test whether the QTL fall in functionally enriched genomic regions. We document a polygenic architecture behind the song rhythm divergence in the inter-island species pair that is remarkably similar to that previously found for an intra-island species pair in the same genus. Importantly, the QTL regions were significantly enriched for potential homologs of the genes involved in pathways that may be modulating the cricket song rhythm. These clusters of loci could constrain the spatial genomic distribution of the genetic variation underlying the cricket song variation and harbor several candidate genes that merit further study.

## 1. Introduction

Behavioral divergence can produce significant reproductive barriers in animals and can be an important force driving speciation [1,2,3,4,5]. However, the genetic mechanisms leading to behavioral divergence, suppression of interspecific recombination, and ultimately the origin of a new species remain poorly understood [3,6,7,8,9]. One way to advance our understanding of the speciation process and the evolution of behavioral divergence is to describe and compare the genetic architectures—the number of genes, their effect size, and their genomic distribution—underlying behavioral isolation in replicate species pairs that diverge in the same phenotype. In doing so, we can gain insight into the genetic causes and consequences of divergence in important speciation forces and phenotypes.

The traits involved in behavioral isolation are often quantitative, and the divergence in these traits is usually caused by many changes of small effect [3,6]. However, it has been argued that the type of genetic architecture, specifically whether phenotypic change is the result of polygenic, additive alleles (Type I) or of major effect alleles with strong epistatic modifiers (Type II) [10], can be informative about the tempo and mode of speciation [11,12]. All else being equal, a genetic architecture characterized by few alleles of major effect would allow for rapid phenotypic evolution, because the phenotypic effect of a single mutation is expected to be larger; theoretical models for speciation by sexual selection often assume such simple genetic architectures [13,14,15,16]. Moreover, it has been suggested that Type II architectures may be lynch pins in the radiations involving founder effects [11], such as those accompanying the colonization of archipelagos, because of the volatile evolutionary effects of epistatic interactions under fluctuating demography; founder effect speciation has received mixed support theoretically and empirically [17]. Importantly, empirical studies show that divergent sexual traits, which can be an important cause of rapidly evolving behavioral isolation [4], are often associated with Type I genetic architectures [3,18], without a disproportionate contribution from sex-linked loci [3,19] (albeit, the role of X-effects in the evolution of secondary sexual characters remains somewhat contentious, e.g., [20]). A Type I architecture would allow for gradual (but not necessarily slow) and orchestrated divergence of male and female sexual traits due to the predictable, biometrical action of genetic variants on diverging traits [21,22]. However, few studies have directly compared the genetic architecture of the behavioral barriers arising in different biogeographic contexts, for example, by addressing the question of whether the divergence in sexual signaling phenotypes between replicate species pairs diverging within the same island or on different islands is associated with similar genetic architectures.

Other genomic influences may also affect the evolution of reproductive barriers. The number of independent genetic factors and the rate of the phenotypic evolution can be strongly affected when causal genes cluster in specific genomic regions. Fisher [23] suggested that genes associated with the gradual adaptive change of complex phenotypes will become physically linked over time. He imagined that this linkage would maintain the identity of groups with complex phenotypic differences (e.g., divergent populations and dimorphic sexes) by reducing the disruptive effects of recombination in the region with which the phenotype is associated. Indeed, linkage disequilibrium within tight clusters of causal genes can be maintained by low recombination, for example, because of chromosomal inversions [24]. Additionally, when populations diverge in the face of gene flow, the interacting effects of the gene flow and divergent selection may favor co-adapted gene complexes to become linked in regions of low recombination [25], as may be the case in, for example, stickleback [26]. Alternatively, (tight) physical linkage of multiple genes that jointly affect the same phenotype could also constitute a gene cluster [27]. The involvement of gene clusters has been implicated in the rapid evolution of (co)adaptive phenotypes in plants and animals [28], including courtship behavior [29,30,31,32]. Such clusters allow for orchestrated adaptive responses by reducing recombination between co-adaptive alleles and may be an important mechanism underlying the natural variation in complex traits, such as altruistic signals (i.e., ‘green beard’ phenotypes) [33], behavior, and co-evolving sexual signals and preferences [22,23,34].

Here, we examine the genetic basis of sexual trait divergence in the rapidly diversifying, endemic Hawaiian cricket genus, *Laupala.* We test whether the biogeographic context of speciation can predict the genetic architecture of courtship song divergence and whether the distribution of the causal loci is potentially constrained by the clustering of functionally related genes. Differentiation in acoustic sexual signaling behavior appears to play a central role in *Laupala* speciation, a group with one of the highest speciation rates documented for arthropods [35]. Species are endemic to single islands and show striking differentiation in male song and female (song) preference [35,36]. Like most other cricket species, the male produces a song by rubbing together its specialized forewings. The songs of *Laupala* crickets are relatively simple and consist of trains of pulses, each pulse produced by a single closing movement of the wings. Evidence suggests that a critical trait used in female mate choice is the repetition rate of these pulses (i.e., the pulse rate [37,38]), which is highly heritable and constitutes one of the major sources of phenotypic divergence among the *Laupala* species [39,40,41,42,43].

So far, we have not been able to pin cricket song rhythm variation down to individual genes. However, the neurobiology of the cricket song generation is generally well understood, involving contributions from descending brain neurons, specialized motor neurons in the thorax called central pattern generators, and related neuromuscular modulators such as ion channels and synaptic targets [44,45,46,47,48]. Therefore, other organisms with known genetic and neurological pathways that drive sex-specific and (acoustic) courtship behaviors can shed light on the potential mechanisms at play in *Laupala* song divergence. In *Drosophila melanogaster*, sexual dimorphism in the nervous system, driven by the interaction between the transcription factors *doublesex* and *fruitless*, provides a developmental foundation for the courtship song [49,50]. However, neither of these genes contributes to interspecific differences in song rhythm [51]. Other genes, including *slowpoke* and *cacophony*, have been implicated in species and population differences across *Drosophila* species: these genes control the properties of the ion (calcium and potassium) channels at neuromuscular junctions [52,53,54,55] and can transform the output of neuronal networks including central pattern generators [56]. Therefore, we may expect similar neuromodulators as well as genes involved in the synaptic transmission at the neuromuscular junctions and rhythmic behavior to contribute to variation in song rhythm in crickets.

The first goal of this study is to illuminate the genetic architecture of sexual trait divergence in light of different geographic modes of speciation. Despite rapid phenotypic divergence between *Laupala cerasina* and *Laupala eukolea* following the colonization of a new island [57], evidence from a previous biometric study shows that multiple genetic factors (~5) underlie the pulse rate variation between these species [43]. We build on this knowledge using quantitative trait locus (QTL) mapping to examine the number, effect size, genomic distribution, and interactions of the loci contributing to the interspecific pulse rate variation between *L. cerasina* and *L. eukolea*, in order to characterize the genetic architecture as polygenic and mostly additive (Type I), or one of major effect loci with strong epistasis (Type II). We compare these results to the polygenic genetic architecture known from the independently evolving species pair, *Laupala kohalensis* and *Laupala paranigra,* which diverged in pulse rate within a single island, that is, Hawaii [35,36,58]. Although *L. cerasina* (Hawaii Island) likely arose as a consequence of an interisland speciation event from the ancestral source range (Maui) of *L. eukolea* [57], we hypothesize a Type I genetic architecture, based on previously published biometrical results [43]. 

The second aim of this study, inspired by theoretical predictions [23] as well as recent findings on the genetics of mating behavior [29,30,31], is to test the hypothesis that functionally related genes cluster in QTL regions. Using genome-wide, functional genetic data from both sexes, and across ontogenetic stages and reproductive states, we assemble and annotate the *L. cerasina* transcriptome to assign putative gene function to loci in the linkage map, and perform gene set enrichment analysis. Based on neurobiological insights into cricket song generation and neurogenetic insight into song variation in *Drosophila*, we expect QTL regions to be enriched for genes with putative functions in neuromuscular processes associated with song production, rhythmic behaviors, or mating behavior. This would suggest that ‘pools’ of functionally related genes associate with QTL regions, as opposed to QTL regions harboring single genes.

## 2. Materials and Methods

*L. eukolea* nymphs were collected in 2012 in Kipahulu Valley on Maui at Ginger Camp (20°41′60.000″ N; 156°5′18.000″ W) and Palikea Peak (20°40′20.640″ N; 156°4′5.160″ W); *L. cerasina* were collected in Kalopa State Park (20°2′13.200″ N; 155°26′36.960″ W) on Hawaii Island. The animals were kept in the lab in plastic cups under constant temperature (20 °C) and humidity, and were provided cricket chow (Fluker Farms, Port Allen, LA, USA) ad libitum, as well as substrate to lay eggs. Males and females were kept separately to ensure the virginity of all animals. They were phenotyped between three and ten weeks after the final molt, during which time the sexual receptivity is maximized in *Laupala* [42]. Two families of first generation interspecific hybrids (HC1 and HC2) were each generated by mating a *L. cerasina* male with a *L. eukolea* female. Several males and females from each family (14 full sib pairs from each of the families) were used to obtain the second generation hybrids.

The male songs were recorded following the methods in Shaw (1996) [39]. A single recording for each individual (*L. cerasina*, *n* = 24; *L. eukolea*, *n* = 16; F2, *n* = 230) was made between 10 a.m. and 4 p.m. Virgin, adult males were recorded individually in a plastic container with screen covers in an anechoic and temperature-controlled chamber, using a SONY Pro Walkman cassette recorder and SONY microphone. The songs were then digitized using SOUNDSCOPE/16 (GWI Instruments, Cambridge, MA, USA) at 44.1 kHz to generate an oscillogram displaying trains of pulses (singing bouts). We estimated the pulse rate by averaging the inverse of five pulse periods (measured from the onset of a pulse to the onset on the next pulse) measured from a single singing bout.

We extracted DNA from whole adult male crickets using the DNeasy Blood and Tissue Kits (Qiagen, Valencia, CA, USA). The genotype-by-sequencing library preparation and sequencing were done in 2014 at the Genomic Diversity Facility at Cornell University following [59]. The *Pst* I restriction enzyme was used for sequence digestion and was DNA was sequenced on the Illumina HiSeq 2000 platform (Illumina Inc., San Diego, CA, USA) with 100 bp single end reads.

The reads were trimmed and demultiplexed using Flexbar v2.5 [60] and then mapped to the *L. kohalensis* de novo draft genome using Bowtie2 v2.2.6 [61] with default parameters. We then called single nucleotide polymorphisms (SNPs) using two different pipelines, The Genome Analysis Toolkit v3.6.0 (GATK) [62,63] and FreeBayes v0.9.13 [64]. For GATK we used individual BAM files to generate gVCF files using ‘HaplotypeCaller’, followed by the joint genotyping step ‘GenotypeGVCF’. We then evaluated the variation in SNP quality across all of the genotypes using custom scripts in R v3.3.1 [65] to determine the appropriate settings for hard filtering using the following metrics, based on the recommendations for hard filtering [66]: quality-by-depth, Phred-scaled *p*-value using Fisher’s exact test to detect strand bias, root mean square of the mapping quality of the reads, u-based z-approximation from the Mann–Whitney rank sum test for mapping qualities, u-based z-approximation from the Mann–Whitney Rank Sum Test for the distance from the end of the read for the reads with the alternate allele. For FreeBayes, we called variants from a merged BAM file using standard filters. After the variant calling, we filtered the SNPs using ‘vcffilter’, a Perl library part of the VCFtools package [67] based on the following metrics: quality (>30), depth of coverage (>10), and strand bias for the alternative and reference alleles (SAP and SRP, both >0.0001). Finally, the variant files from the GATK pipeline and the FreeBayes pipeline were filtered to contain only biallelic SNPs with less than 10% missing genotypes, using VCFtools v0.1.15. We retained all of the SNPs that had identical genotype calls between the two variant discovery pipelines. We then pruned our data further to contain only ancestry informative markers (i.e., grandparents are homozygous for alternative alleles), one SNP per scaffold, markers with no or only limited segregation distortion from the expected 1:2:1 (autosomal) and 1:1 (X-linked) ratios (χ^2^ associated *q*-value ≤ 0.05, i.e., a 5% false discovery rate, [68]), and markers with fewer than 99% of their genotypes in common with other marker loci (i.e., exclude one of each pair of marker loci with identical genotypes for all individuals).

For each of the hybrid cross families, HC1 and HC2, we created linkage maps separately using MapMaker v3.0b [69], taking the following three steps. Firstly, markers were grouped into linkage groups using the ‘group’ command with the ‘default linkage criteria’ set to 4.0 LOD (logarithm of the odds) and 35 centimorgan (cM). Secondly, for each group, a subset of the marker passing a series of quality criteria (i.e., informative, well-spaced markers) were ordered using the ‘order’ command. The informative markers were those with no missing genotypes and that were more than 2.0 cM apart from other markers. The marker order was compared with regression mapping in JoinMap v4.0 [70] and the inconsistencies were resolved by minimizing the stress (in JoinMap) and map length, and maximizing the likelihood (in MapMaker). Thirdly, the remaining markers were added with the ‘build’ command and the order was verified using the ‘ripple’ command. At this step, the markers were added to the map, satisfying a log-likelihood threshold of 4.0 for the positioning of the marker (i.e., the assigned marker position is 10,000 times more likely than all of the other positions), then adding the remaining markers at a log-likelihood threshold of 3.0, followed by a final addition at a log-likelihood threshold of 2.0. Subsequently, any unincorporated markers were discarded. To determine the final marker order we used the ‘ripple’ command with a window size of six markers and a log-likelihood threshold of 2.0. The arbitrary orders in marker dense regions (i.e., orders with similar likelihoods) were resolved using information from both HC1 and HC2 maps, choosing the order that maximized the likelihood and minimized the map length (measured in cM) for both cross families. 

Finally, we merged the separate HC1 and HC2 maps using the R package LPmerge v1.6 [71]. LPmerge uses linear programming to combine two maps from independent populations, based on the similarities in the marker order. Incongruent marker orders between HC1 and HC2 (i.e., linear inequalities) were solved based on the weight assigned to each independent linkage map. The solution also depended on the size of the interval, *K*, in which the conflicting markers were detected and re-ordered (or removed if no solution was found, removing a constraining marker improved the linear equality). For each linkage group, separately, we varied the weighting of the two linkage maps and the interval in which the linear inequality was resolved (*K*) in order to find the consensus map associated with the lowest mean and the variance of the root-mean-squared error between the consensus map and the original maps. 

We used composite interval mapping (CIM) and multiple-QTL models (MQM) in the R/qtl v1.42 [72] package to detect and locate QTL and to calculate the effect sizes independently for HC1, HC2, and for the merged (consensus) map. We first performed a single QTL scan using the ’scanone’ function with the multiple imputation method [73] and the Haley–Knot regression [74]. For CIM, we then ran a model using the Haley–Knot regression in 20 cM windows, with the number of included marker covariates dependent on the number of QTL detected in the single QTL scan. We then performed a two-dimensional (2D) QTL scan using the Haley–Knott regression to detect pairs of QTL and the interaction effects among QTL and permuted the two-dimensional QTL to establish the penalized likelihood criteria for the main and interaction effects. We subsequently built a multiple QTL model, starting with the QTL, with the highest LOD score in the single QTL scan, refining the position using ‘refineqtl’, and then scanning for additional QTL. We continued adding QTL (followed by refining their position) until an additional QTL did not improve the LOD score of the model beyond the penalized LOD score threshold for the main effects at the α = 0.05 level. After the addition of each QTL, we checked for potential QTL interactions that would improve the multiple QTL model beyond the (heavy) penalized LOD score threshold for interaction effects at the α = 0.05 level. Finally, we estimated the effect sizes by fitting the QTL model and using the drop-one-term analysis. In the merged map, we included the cross type as a covariate in all of the steps described above.

To estimate the true number of genetic loci underlying the pulse rate variation based on the QTL results, we use the method of Otto and Jones [75]. We estimated the minimum detectable QTL effect size using Equation (11) in Otto and Jones [75], specifying *a_min_* as the smallest QTL, we detected in the QTL scan (for HC1, HC2, or for the combined map). We then used Equation (6) in the work of Otto and Jones [75] to estimate the true number of loci. The calculations were done using custom functions in R.

To look for candidate genes in the QTL regions and to test for the enrichment of specific gene functions, we first assembled the transcriptome to obtain information about the putative gene function of the loci within the regions of interest. First generation, lab-reared, whole *L. cerasina* individuals were used for RNA-sequencing and transcriptome assembly. A total of ten samples were stored in RNAlater, following the manufacturer’s recommendations (Qiagen, Valencia, CA, USA), and were pooled by sex prior to sequencing, as follows: four adult males (two sampled in the morning and two sampled in the evening, one of which was sampled immediately after mating), four adult females (likewise, two were sampled in the morning and two sampled in the evening, one of which was sampled immediately after mating), and a juvenile male and female. We sampled both of the sexes, the adult and juvenile life stages, and individuals of variable mating status to account for the differential expression among such individuals, specifically when the genes are only expressed in one of the sexes or only in the adults or juveniles. The tissue was homogenized using sterilized forceps in RNAlater. The RNA was extracted using the RNAeasy kit (Qiagen, Valencia, CA, USA). A quality check was done using a NanoDrop spectrophotometer (Thermoscientific, Wilmington, DE, USA) and the Agilent Bioanalyzer 2100 (Agilent Technologies, Santa Clara, CA, USA) [76]. The samples were then sequenced on a single lane on the Illumina HiSeq 2000 platform, with 50 bp paired-end reads. The reads were processed using Fastq-mcf from the Ea-Utils package [77] with the parameters -q 30 (nucleotides from the extremes of the read with qscore below 30 were trimmed) and -l 30 (reads with lengths below 30 bp discarded). The read duplications were removed using PrinSeq v0.20.4 [78] and the reads were corrected using Musket v1.0.8 [79] with the default parameters.

We assembled the *L. cerasina* transcriptome using Trinity’s v.2.4.0 [80] genome-guided assembly pipeline. We used the *L. kohalensis* reference genome [81] and a maximum intron size cut-off of 5000 bp. We first created a HISAT v4.8.2 [82] index of the genome and then aligned the male and female paired-end reads to the genome using the default settings. We sorted the resulting alignment SAM file and converted it to BAM format using Samtools v1.5 [83]. We then did the genome-guided assembly using Trinity and checked the quality of the assembly by calculating the N50 statistics, mapping the male and female reads back to the transcriptome using Bowtie2, and searching for conserved eukaryote and arthropod genes from the BUSCO database v.2.0.1 [84]. We then mapped the reads back to the transcriptome using GMAP v 2017-05-08 [85] and BLAT v35x1 [86], and used custom R scripts to retain a single best hit scaffold for each transcript, based on the coverage, identity, and number of matched bases. 

We checked for and removed contaminants using NCBI’s VecScreen, using the UniVec Core database and the recommended BLASTn parameter values [87]. We then functionally annotated the transcripts in three steps using BLAST [88,89], as follows: Firstly, we matched our transcripts against the *D. melanogaster* proteome using an e-value cut-off of 1 × 10^−5^. Any transcripts that were not assigned a putative *D. melanogaster* homolog were matched (at the same threshold) with the Uniprot/Swissprot data base [90], limiting our search to arthropod proteins. Finally, we matched any remaining transcripts after the second step against the non-redundant protein database at NCBI, with an e-value cut-off of 1 × 10^−5^ and limiting our search to animal proteins. 

We then used hmmer2go v3.1 (https://github.com/sestaton/HMMER2GO) to estimate the open reading frames (ORFs) and translated only a single, longest ORF per transcript. We annotated the retaining protein sequences using InterProScan v5 [91,92]. We imported the transcriptome FASTA file, the XML output from the BLAST searches, and the InterPro results into Blast2Go v4.1.9 [93]. We recovered the original BLAST best hit and ran the Gene Ontology mapping using the default settings. We then merged all of these results and ran the Annotation tool.

If the genes controlling pulse rate variation in *Laupala* are clustered in specific genomic regions rather than distributed randomly across the genome, we expect the QTL regions to contain several (putative) causal genes for interspecific pulse rate variation. We first used topGO v2.24.0 from R’s BioConductor v3.3 environment [94] for the gene set enrichment analysis. We combined all of the transcripts matching the scaffolds within the peak ±1 LOD interval for each of the seven QTL peaks in the consensus linkage map (i.e., scaffolds with a marker linked to pulse rate variation at a likelihood of no less than the peak LOD score minus 1). We then used the parent-child *p*-value correction [95] and an additional false discovery correction [68] with the ‘p.adjust’ function in R (the genetic ontology (GO) terms were considered enriched at a false discovery rate of 10% or less). As these analyses are potentially confounded by pseudo-replication in the transcriptome assembly (e.g., because of varying occurrences of exonic splice sites and transcription start sites), we perform the above analysis after collapsing all of the transcripts that were mapped to the same scaffold and were considered a putative alternative splice form based on their annotations (i.e., transcripts that have the same predicted gene product, are annotated with different isoforms of the same gene or partial and full hits of the same gene). For comparison, we also conduct the GO enrichment analysis before collapsing the pseudo-replicates. Finally, we manually inspected the putative gene function in the QTL regions by examining the experimentally proven biological and molecular functions for the highest ranked BLAST annotations on FlyBase [96].

## 3. Results

### 3.1. Linkage Mapping

We mapped a total of 298 and 416 markers for HC1 (*n* = 94) and HC2 (*n* = 136), respectively, to eight linkage groups corresponding to the seven autosomes and the X-chromosome in *Laupala* (Figure S1). The map lengths were 821.2 cM and 734.1 cM, corresponding to 2.72 cM and 1.76 cM, per marker, respectively. Merging the maps resulted in 508 unique markers at a total map length of 776 cM (1.52 cM/marker). The maps are broadly similar (in order and length) across the two mapping families. One linkage group, LG1, shows substantially higher recombination rates among the markers in HC1 relative to HC2 (Figure S1), which may be due to sampling variance, structural variation, or both. Comparing the marker order on LG1 with the homologous LG in two interspecies crosses [81] revealed that this region is inverted in the map for HC1 (Figure S2), suggesting that a large pericentric inversion is segregating in either *L. cerasina*, *L. eukolea*, or both. However, without further evidence from, for example, long-read sequencing data, we treat this inversion as putative. 

### 3.2. QTL Mapping

**Figure 1:**
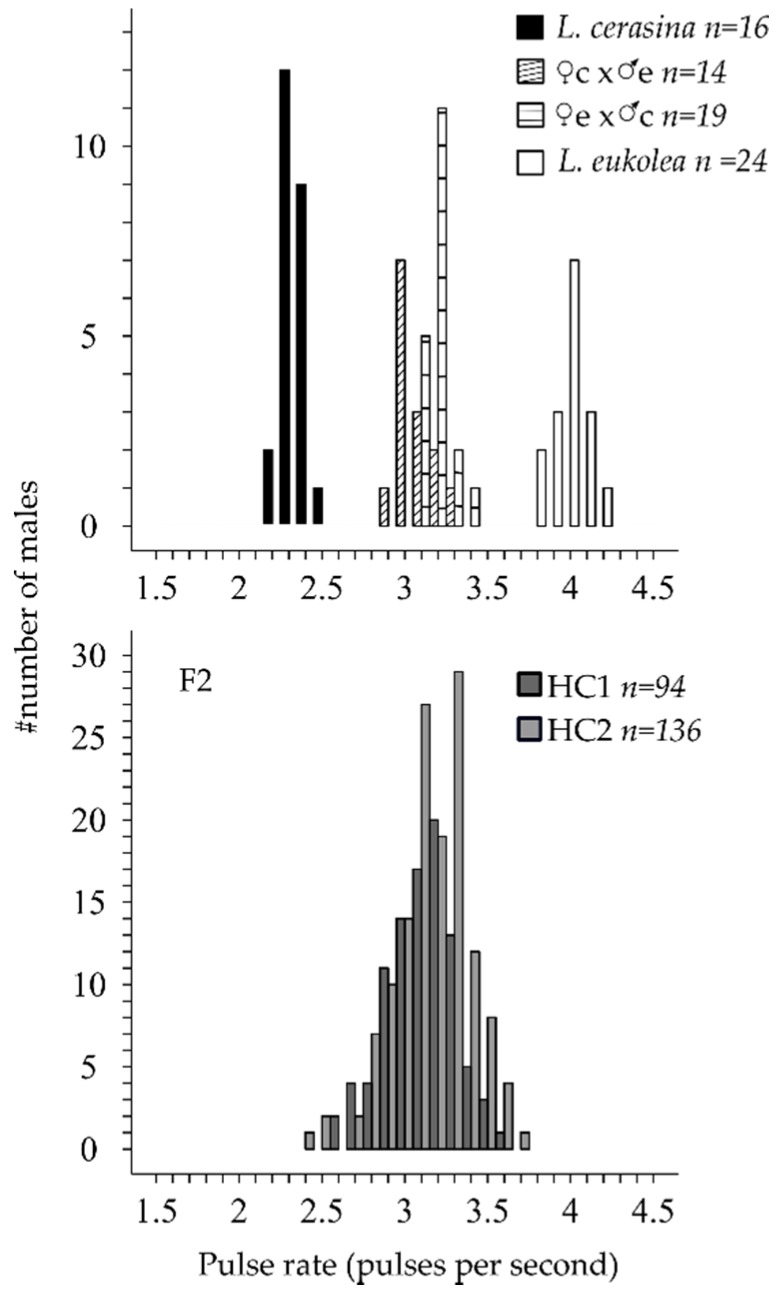
Phenotypic distributions for the parental lines and first- and second-generation hybrid offspring. The data from the F_1_ hybrids in the top panel are from Oh et al., 2012 [43], and are shown to illustrate the F_1_ distributions as well as the maternal bias in the pulse rate inheritance. The F_2_ data are split by cross type (HC1 and HC2).

*L. cerasina* and *L. eukolea* males have non-overlapping, normally distributed pulse rate distributions (Figure 1). The mean pulse rate difference was 1.66 pulses per second (pps; Table 1). The F_2_ song phenotype was normally distributed and the means of the HC1 and HC2 progeny did not differ significantly (t = −1.8046, *df* = 228, *p* = 0.0724). Considering the joint F_2_ distribution, the slow tail of the distribution partially overlapped with the *L. cerasina* phenotypic distributions, although the fast tail did not overlap with the *L. eukolea* phenotypic distribution (Table 1, Figure 1).

**Figure 2:**
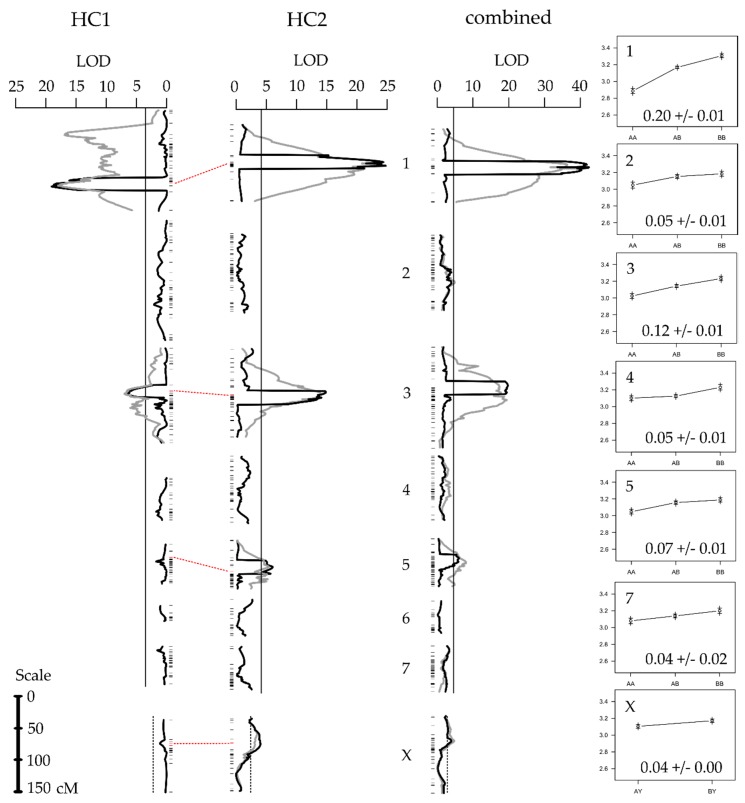
Quantitative trait locus (QTL) scan. Results from composite interval mapping (black lines) and multiple QTL models (grey lines, only for linkage groups with significant QTL). The vertical solid and dotted lines show the experiment-wide 5% significance threshold for the composite interval mapping (CIM) for autosomes and the X-chromosome, respectively. Between HC1 and HC2, the horizontal dotted lines connect the homologous markers associated with the QTL peaks (within the CIM windows) to indicate the overlap between the QTL scans in the different mapping families (see Figure S1 for more detail). The panels on the far right show the effect size of each of the QTL as the pulse rate mean ± standard error for each of the genotype categories AA (left), AB (center), and BB (right).

Using CIM and MQM, we detected two moderate effect (~10% of the species difference) QTL on LG1 and LG3 (Figure 2, Table 2) in both the HC1 and HC2 mapping populations. We note that the LOD profile for MQM in HC1 suggests an additional peak on the LG1 (at 37 cM), but neither CIM nor MQM supports this. We believe the ‘phantom’ peak derives from the putative inversion creating genotype-phenotype associations for markers outside the QTL region in some individuals, but not in others. In HC2, we additionally detected two smaller effect QTL (<5%), on LG5 and LGX. Merging the maps and combining the sample sizes also revealed the small effect of QTL on LG2, LG4, and LG7 (Table 3), using MQM (Figure 2). When adding these additional QTL to the MQM model, the peak on LG3 shifts approximately 10 cM posteriorly, but the 1-LOD intervals of the former and refined QTL overlap (Figure S1), and consequently does not impact our annotations (see below).

All of the (haploid) QTL effects were significantly larger than zero (*p* < 0.05) and of the same sign (Table 2 and Table 3). Together, the seven QTL for the combined HC1 and HC2 mapping populations explained 35.41% (or 0.59 pps) of the haploid phenotypic difference between the parental lines (Table 3; 20.22% and 31.71% for HC1 and HC2, respectively, Table 2). None of the QTL had significant dominant effects (Table S1) and no interactions between additive QTL were detected, thus the total amount (twice the additive haploid effect) of the pulse rate difference explained by the seven QTL is 70.82% (or 1.16 pps) of the species difference. The QTL on LG1 and LG3 was mapped to approximately the same location in HC1 and HC2, with the marker nearest to the peak in HC2 directly flanking the peak marker in HC1 (Figure 2, Table S2).

Based on the MQM results for HC1 and HC2, and using the method in the work of Otto and Jones [75], we estimated the true number of loci to be 9.55 (95% confidence interval = [1.59–29.49]; detection threshold, θ = 0.08 pps) and 9.4 (95% confidence interval = [2.93–21.86]; θ = 0.03 pps), respectively. Using all of the 230 F_2_ individuals, we estimated the true number of loci at 16.6 (95% confidence interval = [7.14–32.12]; θ = 0.04 pps). The variation in the true number of loci between HC1, HC2, and the combined sample, reflects the variation in the mean across all of the detected QTL effects, as well as in the lowest detected QTL effect, which is dependent on the sample size.

### 3.3. Transcriptome Assembly and Annotation

We used the genome-guided assembly from the Trinity pipeline [80] to assemble the transcriptome. Of the 50,148, and 157 reads after filtering (52,980, and 661 prior to filtering) that were used to assemble the transcriptome, 90.58% mapped to the *L. kohalensis* reference genome. The assembly had a total length of 53,928, and 392 bp, the median contig length was 397 bp, and the contig N50 was 1805 bp. The mean coverage was 49.9× and 40.8× for the female and male reads, respectively. The male and female reads mapped back to the transcriptome with high confidence, at mapping rates of 92.05% and 92.26%, respectively. The BUSCO analysis indicated that we captured a large proportion of conserved eukaryote and arthropod genes with 97.4% and 95.2% complete BUSCO hits, respectively. Using the *D. melanogaster* proteome, the arthropod specific Uniprot/Swissprot database and NCBI’s non-redundant database of animal proteins, we successfully annotated 19,713 transcripts (32.2% of all of the transcripts) at a combined length of 32,039, and 513 bp (59% of the full assembly). A total of 17,577 transcripts have a gene ontology (GO) annotation.

### 3.4. Gene Set Enrichment Analysis

**Figure 3:**
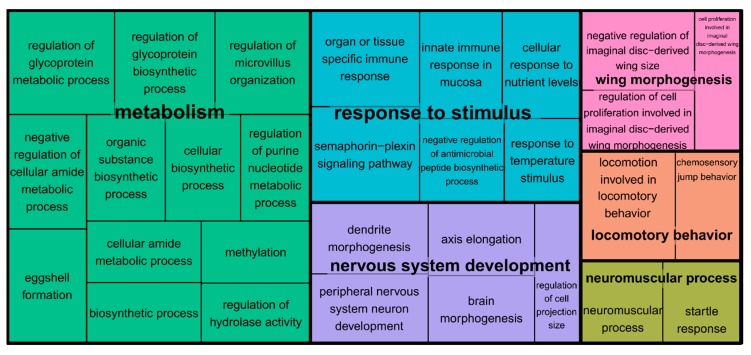
Treemap of enriched genetic oncology (GO) categories. The GO terms were subset, removing all of the redundant GO terms in REVIGO [98] at the medium-similarity criterion (0.7). The GO terms are grouped based on the taxonomic relations among them. The colors connect the GO terms belonging to the same cluster. The size of the panels scales with the negative 10-logarithm of the *p*-value for the enrichment test.

There were 179 scaffolds within one LOD of the seven QTL peaks combined. After collapsing the putative alternative splice forms of the genes (transcripts on the same scaffold with identical predicted protein products or annotated with different isoforms of the same gene), we mapped a total of 1298 annotated transcripts to 171 of the 179 scaffolds (Table S3). We tested whether the QTL regions were significantly enriched for the biological processes that are relevant to cricket mating behavior, that is, sexual (acoustic) communication, muscle contraction and pacemaker genes, and various neuromuscular properties and neuromodulators of rhythmic behaviors. We found a significant false discovery rate (<10%) enrichment of 37 biological processes, many of which are related to neurobiological and muscular development, that is, peripheral nervous system development, dendrite guidance, brain morphogenesis, and neuromuscular junction development in the combined set of all of the seven QTL regions (Figure 3 and Table S4). Similarly, for each of the seven QTL regions separately, we find a significant enrichment of central complex and motor neuron development (LG1), hormonal and pheromonal biosynthetic pathways (LG2), neurotransmitter transport and mating behavior (LG3), peripheral nervous system development (LG4), flight and locomotor behavior (LG5), neuromuscular junction development (LG7), and calcium ion transport (LG X), among others (Table S5). Using the 1-LOD interval for the QTL on LG3 prior to the 10 cM shift in the QLT peak, gave identical results for the enrichment of that QTL region. Excluding the QTL on the LG7, which had a large confidence interval (and only weak phenotypic effect), also gave similar results; although, the locomotory behavior is no longer significantly enriched (data not shown).

When putative alternative splice forms are not collapsed into a single annotated gene product (i.e., the original annotation of the transcriptome assembly), 1724 annotated transcripts map to the scaffolds within the seven QTL intervals (Table S6). We find substantially more enriched GO terms (227 biological processes). However, many of the same terms are enriched compared to the analysis above, both for the overall enrichment (Table S7), as well as for the linkage groups separately (Table S8): for example, terms related to neuromuscular development (LG1, LG4), development, maintenance, and transmission at synapses (LG1, LG3, LG5, and LGX), rhythmic and locomotor behaviors (LG4, LG7), mating behavior (LG3), hormone and pheromone production (LG2), and nervous system development (LG4, LGX; Table S8).

## 4. Discussion

Behavioral isolation is an important barrier to gene flow in the earliest stages of animal speciation [4], but we know little about the number and distribution of the underlying genetic loci and their relationship with the tempo and mode of evolution. Particularly, it has remained understudied whether the divergence in the same phenotype in replicate species pairs diverging in different biogeographic contexts is associated with similar genetic architectures. For example, in island systems where founder effects have been hypothesized in the history of species radiations, shifts in the phenotypic and genetic environments may catalyze speciation through the interactions of genetic drift and the genetic architecture of traits [11,17], in particular, for the traits involved in sexual isolation [17,99,100]. The presence of epistatic, major effect loci (a Type II genetic architecture) may further amplify the effects of genetic drift on phenotypic evolution when the genetic system becomes reorganized during the genetic upheaval following a founder event, and thus acts as a lynch pin for speciation [17].

Here, we show that the genetic architecture of a major premating barrier in *Laupala* speciation following island colonization has a polygenic (Type I) genetic architecture, as is common for sexual signals [3], rather than a Type II genetic architecture that can promote founder effect speciation. This finding, compared to the previous study of an intra-island divergence event, indicates that similar genetic architectures underlie repeated episodes of mating song divergence in *Laupala*, independent of the biogeographic history. However, further study is required to determine whether divergence in the sexual signaling phenotypes in the replicate species pairs of *Laupala* involve the same QTL, because we are currently limited to making comparisons between QTL experiments using different marker types (GBS markers versus Amplified Fragment Length Polymorphism (AFLP) markers) constraining the resolution of the comparison to the linkage group level. We also find that the QTL regions co-localize with groups of genes that are enriched for several interesting biological processes. This provides a tentative explanation for the similar genetic architectures in replicate species pairs (if the same QTL regions are involved). These putative gene clusters in the QTL regions also suggest that a large pool of genes and numerous functional sites could potentially contribute to the song evolution in *Laupala*, thus restricting the spatial genomic regions of phenotypic change, but not the number of quantitative trait nucleotides. Lastly, we identify many putative *Laupala* homologs of several genes implicated in *D. melanogaster* courtship behavior, and in various neurophysiological processes that might be important for song rhythm divergence in *Laupala*.

### 4.1. The Genetic Architecture of Interspecific Pulse Rate Divergence

Our data support the hypothesis that the song divergence between *L. eukolea* and *L. cerasina* is associated with a Type I genetic architecture. We detected seven small-to-moderate effect QTL for pulse rate divergence, six QTL each on different autosomes and an additional small effect X-linked QTL. There were no detectable interaction effects among these identified QTL. The position and effect size of the two largest QTL between the replicate families were largely the same (shared scaffolds on peak or flanking [or both] markers on LG1 and LG3; Table S2). The effects of the sample size being well-known [101], we merged the families to leverage the power of the increased sample size. We found that the two QTL detected in HC2 but not in HC1 (on LG5 and LGX) shared common positions and effect sizes with this combined analysis (Table S2). In the combined analysis, we further detected three small effect QTL on LG2, LG4, and LG7. Adding all of the QTL terms to the MQM model resulted in a refined estimate of the peak QTL location on LG3 in the combined map. Further exploration revealed that this position refinement was not specific to the map used in the analysis, as simulated QTL experiments as well as adding the additional QTL (that are borderline significant or only ‘suggestive’) to the HC1 and HC2 models yield similar effects (not shown). Drawing on all of the QTL identified, and the method of Otto and Jones [75], we estimate that the true number of loci is likely more than ten. 

### 4.2. Inter versus Intra-Island Speciation

Contrasting the results for *L. cerasina* and *L. eukolea* with those for the intra-island species pair *L. kohalensis* and *L. paranigra* [58] suggests that the differentiation of song has occurred by similar genetic architectures in these independent divergence events; we detect similar numbers of loci on the same linkage groups, with comparable effect size distributions (Table 4). Morphological [36] and molecular evidence [35] place the replicate species pairs considered above in independent species groups. Based on the young age of the Big Island [102], to which *L. kohalensis, L. paranigra*, and *L. cerasina* are endemic, both of these species pairs have likely diverged in the last 500,000 years [35], but differ in the biogeographic context of speciation. We estimate that the two major QTL, on LG1 and LG3, explain around 12% and 8% of the average phenotypic difference between *L. eukolea* and *L. cerasina*. The homologous linkage groups in the genetic map of *L. kohalensis* and *L. paranigra* (linkage group numbers are the same) have QTL that explain around 9% and 10% of the parental difference, respectively. Likewise, both the present study and the *L. kohalensis* and *L. paranigra* cross study found additional QTL on LG4, LG5, and the X-chromosome, with no detected interactions among the loci. Previously, biometric studies had also revealed multiple independent genetic factors and an X-effect [39,43]. Moreover, the phenotype associations on LG6 and LG7 were weak or absent in both of the studies, and, further estimates of the true number of loci are >10 in both of the studies, suggesting a polygenic architecture for both the inter-island and intra-island speciation. Finally, all of the QTL effects that were estimated in this study and in Shaw et al. (2007) [58] are of the same sign, consistent with a hypothesis of directional selection [103]; that is, the alleles from the fast species increase the pulse rate, whereas the alleles from the slow species decrease the pulse rate of the F_2_ hybrids. Thus, overall, these results support similar genetic architectures for pulse rate divergence, regardless of the biogeographic context. We caution, however, that other phenotypes may or may not follow this pattern (e.g., cuticular hydrocarbon variation [104]), and merit further investigation.

There are also some important differences between the analyses of the two species pairs. The pulse rate difference between *L. cerasina* and *L. eukolea* is roughly half of that between *L. paranigra* and *L. kohalensis* [58], which could explain some of the differences in the effect sizes between the two studies (Table 4, see below). We detected just seven QTL in the present study as opposed to eight QTL in the *L. paranigra* and *L. kohalensis* cross, despite similar sample sizes and a much denser genotype sampling in the present work. We also find smaller average effect sizes for the *L. cerasina* and *L. eukolea* QTL. For example, while the QTL effects on LG4 and LG5 are present in both of the species pairs, they are smaller (both in absolute and relative terms) in the *L. cerasina* and *L. eukolea* cross than the *L. kohalensis* and *L. paranigra* cross (0.05 and 0.07 pps versus ~0.14 and ~0.29 pps, respectively). Additionally, we did not find evidence for minor QTL on LG1 and LG3 in the present study, although both linkage groups were found to harbor minor peaks in the 2007 study. It must be noted that the resolution at which we resolve the QTL regions is in the order of several centimorgans. Additionally, Shaw et al. (2007) [58] used AFLP markers while we use GBS markers. Therefore, comparisons of the genetic architectures between these two independent species pairs can only be made at the chromosomal (linkage groups) level, because we lack sequenced-based markers and precise genomic locations of QTL in *L. paranigra* and *L. kohalensis*. More detailed information is needed to test whether independent divergence in pulse rates is associated with convergent genetic mechanisms.

However, the overall similarity in the genetic architecture is significant in that it shows for *Laupala*, which has one of the fastest rates of speciation known, that the genetic architecture of divergence in an important speciation phenotype is independent of the biogeographic context. Moreover, our findings suggest that differences in the extent of phenotypic differentiation are due to the larger effect sizes of the substitutions in the same QTL regions, rather than that additional QTL are involved in the more diverged species pair. Further comparative work is needed to probe the generality of these findings and to better illuminate the relationship between the biogeography, magnitude of phenotypic divergence, and genetic architecture of speciation. Additionally, potential mechanisms that constrain the number of possible locations in the genome where the genetic changes that contribute to song rhythm variation can occur, need to be examined. One potential mechanism could be that the causal loci are not randomly distributed across the genome, but instead, that they cluster in specific genomic regions.

### 4.3. Behavioral Gene Clusters

Clustering of the causal loci that are important to reproductive isolation is expected on both theoretical [23,105] and empirical [29,30,31,32] grounds, and can have dramatic consequences for the mode and rate of evolution. We find evidence for putative gene clusters in the QTL regions associated with the pulse rate divergence in *L. cerasina* and *L. eukolea*. Although currently the causal genes driving pulse rate variation in this system are unknown, we observe a strong enrichment of the gene sets that might contribute to the cricket mating behavior variation. This enrichment thus provides preliminary evidence that the QTL regions co-localize with multiple (rather than single or very few) genes that may contribute to sexual signal evolution in this system. Enrichment is evident for all of the QTL combined, with or without the QTL on LG7 (which has an exceptionally broad confidence interval). Moreover, the pattern is not driven by a single region, but rather, significant enrichment contributions derive from every QTL region separately. Interestingly, some of these QTL fall in regions of low recombination (e.g., QTL on LG1, LG3, and LG5), in the central parts of the chromosomes [81], where we observed high marker densities (Figure S1). Reduced (interspecific) recombination rates can reinforce linkage disequilibrium between the co-adapted loci over larger genomic distances. Together, these findings suggest that acoustic mating behavior divergence in crickets is potentially associated with clusters of causal loci rather than randomly distributed loci.

Overall, the finding that the QTL regions are strongly enriched for homologs of genes involved in neuromodulation and nervous system development is an exciting novelty in the attempt to unravel the genetic architecture of premating isolation in a model system for speciation research. We acknowledge, however, that the evidence for the presence of functional genetic clusters is preliminary. It is not known whether any of the gene products contributing to the enrichment are also involved in controlling the (variation in) cricket song nor whether the effects from multiple or a single substitution(s) amount to the observed phenotypic divergence. Furthermore, the inference of genetic clusters is limited by a number of methodological constraints. Firstly, our annotations are mostly based on *D. melanogaster* proteins, which are rather divergent from crickets, and hence depend on the presence of conserved regions. We therefore have an incomplete identification of the homologs and GO annotations. In addition, we can only annotate scaffolds that are in the linkage map, which are, in each case, inherently a subset of the scaffolds that make up a given genomic region. However, it is not apparent to us that the sampling that we are able to do, while incomplete, would bias our results in favor of the GO enrichment and gene clustering we observed.

The genomic clustering of the causal loci controlling species differences in the pulse rate would have profound consequences on the evolution of *Laupala* mating behavior during speciation. The genomic clustering of genes has been associated with several traits that are important in reproductive isolation [32,106], speciation [107], and mating behavior variation [29,30,31]. Gene clusters would offer a potential adaptation to overcome the constraints associated with behavioral evolution, which surely requires coordinated changes in many of the loci controlling complex neurophysiological traits. A close linkage would reduce interspecific and, potentially, intraspecific recombination, and facilitate co-adaptation [24]. However, it is unlikely that genetic clusters in the system studied here are the result of selection against interspecific recombination or of the interaction between divergent selection and gene flow as *L. cerasina* and *L. eukolea* diverged in allopatry. The linkage of multiple song genes, but also of the song and preference genes, could speed up divergence and speciation [108,109]. In *Laupala*, there is evidence for the co-localization of male song and female preference QTL [110,111]. The linkage and orchestrated evolution of the different song genes and of the song and preference genes might be facilitated by the reduced recombination, and the co-adaptive gene clusters might contribute to the rapid divergence of mating behavior in the young but diverse radiation of *Laupala*.

### 4.4. Candidate Genes

Our results also suggest potential candidate genes that control the mating behavior variation in *Laupala*. The main enriched biological processes among the predicted gene products in the QTL regions can be tied to potential modulators of the central pattern generators (driving rhythmic behaviors) and to the sex-specific expression of the nervous system development pathways in fruit flies. These findings were, in part, driven by the potential homologs of the motor neuron development gene *roundabout* (1.5 cM away from the peak at LG1), of a Leucin-rich repeat kinase involved in the neurodegenerative disease and locomotion located 0.5 cM from the peak on LG3, of *lola* (transcription factor regulating neuromuscular development 6 cM away from the peak at LG4), and of *semaphorin* (directly flanking the peak at LG7). All but the Leucin-rich repeat kinase are affected by the sex-specific transcription of *fruitless* in *D. melanogaster* (located on LG2 in *Laupala*) and contribute to the sexual dimorphism in the nervous system [112,113,114].

In addition to these sex specific receptor proteins, we find receptors for serotonin, GABA, dopamine, and acetylcholine, all known neuromodulators of central pattern generators in insects [47,115,116]. We also identify several ion channel genes, such as *cadherin* (flanking the QTL peak marker on LG3), *KCNQ potassium channel* (1.5 cM from the peak marker on LG3), *cacophony* (16 cM from the peak on LG4, which has a functional role in the inter-pulse interval in *D. melanogaster* [117]), and *sandman* (1.9 cM from the peak at LG5). Without functional evidence, however, we can only consider these genes as candidate loci and cannot speculate further about the genetic and neurobiological pathways involved in song generation and song differentiation in *Laupala*.

## 5. Conclusions

Together, this study presents rare comparative insights into the polygenic genetic architecture associated with sexual trait divergence during speciation in different biogeographic contexts. Clearly, the rapid quantitative trait differentiation associated with speciation can occur under a polygenic genetic architecture, where many genes diverge in concert to produce a conspicuous species difference. We show that the genetic architecture of male song rhythm divergence in closely related *Laupala* species is remarkably similar among the two independently originated species pairs, with comparable QTL numbers, effect sizes, and an overall absence of interaction among the loci, despite their different geographic histories. These similarities may, in part, result from constraints on the spatial distribution of genetic variation controlling pulse rate divergence, as a result of the clustering of causal loci. We show that the identified QTL regions underlying the song divergence are enriched for a variety of neuromuscular processes, potentially contributing to modulating the central pattern generators that control the song rhythm. This enrichment pattern suggests a compelling genetic potential, deriving from the clustering of multiple, physically linked loci, for rapid divergence in *Laupala* mating behavior. We further identify several potential candidate genes controlling a highly divergent behavioral phenotype that forms a major barrier between the recently diverged species.

## Figures and Tables

**Table 1:** Phenotypic distributions. The mean and standard deviation of the pulse rate (pulses per second) and the sample size are shown for the parental species and the F_2_ generation (both cross types, HC1 and HC2).

	Mean (pps)	sd	*n*
*Laupala cerasina*	2.33	0.07	24
*Laupala eukolea*	3.99	0.12	16
Environmental variance *	0.02	0.13	
F_2_ HC1	3.11	0.20	94
F_2_ HC2	3.16	0.23	136
F_2_ mean	3.14	0.22	230

* Environmental variance was calculate following the work of Fishman et al. [97]; ‘mean’ in this context refers to the environmental variance, while ‘sd’ is the square root of that variance.

**Table 2:** Quantitative trait locus (QTL) results from HC1 and HC2. The QTL were mapped using the maps for the 94 HC1 and 136 HC2 F2 individuals. LOD—log-of-odds. The A and B alleles denote *L. cerasina* and *L. eukolea* alleles, respectively. All of the QTL effects (in pulses per second) are significantly different from zero. ESD—environmental standard deviation.

					Phenotypic Value						
Linkage Group	Location (cM)	LOD	Nearest Scaffold	Marker Location	AA	AB	BB	Effect (pps)	% Parental Difference	#ESDs	% F2 Variance
**HC1**											
1	119	17.81	S004794	119.4	2.88	3.14	3.31	0.220	13.23	1.75	50.60
3	72.0	7.04	S001552	71.3	2.95	3.13	3.16	0.116	6.95	0.92	14.96
**HC2**											
1	56.9	23.27	S002490	56.9	2.90	3.18	3.31	0.217	13.04	1.72	38.67
3	65.0	13.92	S000355	67.1	3.03	3.17	3.28	0.148	8.90	1.08	19.42
5	38.4	5.55	S002808	38.4	3.04	3.17	3.25	0.074	4.45	0.60	6.67
X	32.0	3.42	S000108	29.7	3.13	- ^a^	3.19	0.049	2.95	0.39	3.96

^a^ Cricket males are hemizygous.

**Table 3:** QTL results from the combined map. The QTL were mapped using the consensus map for the 230 F_2_ individuals. LOD—log-of-odds. The A and B alleles denote the *L. cerasina* and *L. eukolea* alleles, respectively. All of the QTL effects are significantly different from zero. ESD—environmental standard deviation.

					Phenotypic Value						
Linkage Group	Location (cM)	LOD	Nearest Scaffold	Marker Location	AA	AB	BB	Effect (pps)	% Parental Difference	#ESDs	% F2 Variance
1	59.0	40.73	S001131	58.8	2.89	3.17	3.31	0.203	12.22	1.61	36.39
2	71.0	4.78	S001921	69.1	3.05	3.15	3.18	0.055	3.30	0.44	2.90
3	79.9	21.28	S000385	79.9	3.02	3.14	3.23	0.123	7.40	0.98	15.34
4	59.8	3.97	S016452	59.0	3.10	3.12	3.23	0.053	3.19	0.42	2.39
5	36.9	8.32	S002445	36.9	3.05	3.16	3.19	0.068	4.08	0.54	5.23
7	5.0	3.51	S007011	9.8	3.08	3.14	3.20	0.045	2.68	0.35	2.10
X	38.0	5.46	S003132	37.2	3.10	-	3.17	0.041	2.46	0.32	3.34
cross	-	2.24	-	-	-	-	-	0.092	5.53	0.44	1.33

**Table 4:** Comparison of the QTL effects for intra-island versus inter-island divergence. The QTL effects (in pulses per second and as a percentage of the parental difference) are shown for the Shaw et al. (2007) [58] study (the intra-island comparison, results from Table 2b in that study) and for the results of the present study (the inter-island comparison).

LG	Intra-Island (Shaw et al. 2007 [58])		Inter-Island (This Study)	
	pps	% Parental Difference	pps	% Parental Difference
1	0.281 *	9.3 *	0.203	12.2
2	0.098	3.3	0.055	3.3
3	0.152 *	5.1 *	0.123	7.4
4	0.143	4.8	0.053	3.2
5	0.297	9.9	0.068	4.1
7	…	…	0.045	2.7
X	0.231	7.7	0.041	2.5

* On LG1 and LG3, two peaks were detected in the intra-island comparison. Here, only the major peak is shown.

## Supplementary Materials

The following are available online at http://www.mdpi.com/2073-4425/9/7/346/s1, Figure S1: Linkage map and multivariate QTL model results, Figure S2: Inversion on LG1, Table S1: QTL dominance effects, Table S2: QTL peak and flanking markers, Table S3: Annotated transcripts mapping to QTL regions after correcting for pseudo-replication, Table S4: GO enrichment of transcripts in Table S3, Table S5: GO enrichment of transcripts in Table S3 per linkage group, Table S6: Annotated transcripts mapping to QTL regions without correcting for pseudo-replication, Table S7: GO enrichment of transcripts in Table S6, Table S8: GO enrichment of transcripts in Table S6 per linkage group.
