# Supplementary material for: The Genetics of a Behavioral Speciation Phenotype in an Island System
Source: Genes (Basel). 2018 Jul 10;9(7):346. doi: 10.3390/genes9070346 (PMC6070818; doi:10.3390/genes9070346)
Supplement: Supplementary file 1 [file genes-09-00346-s001.zip › FigS2.pdf]

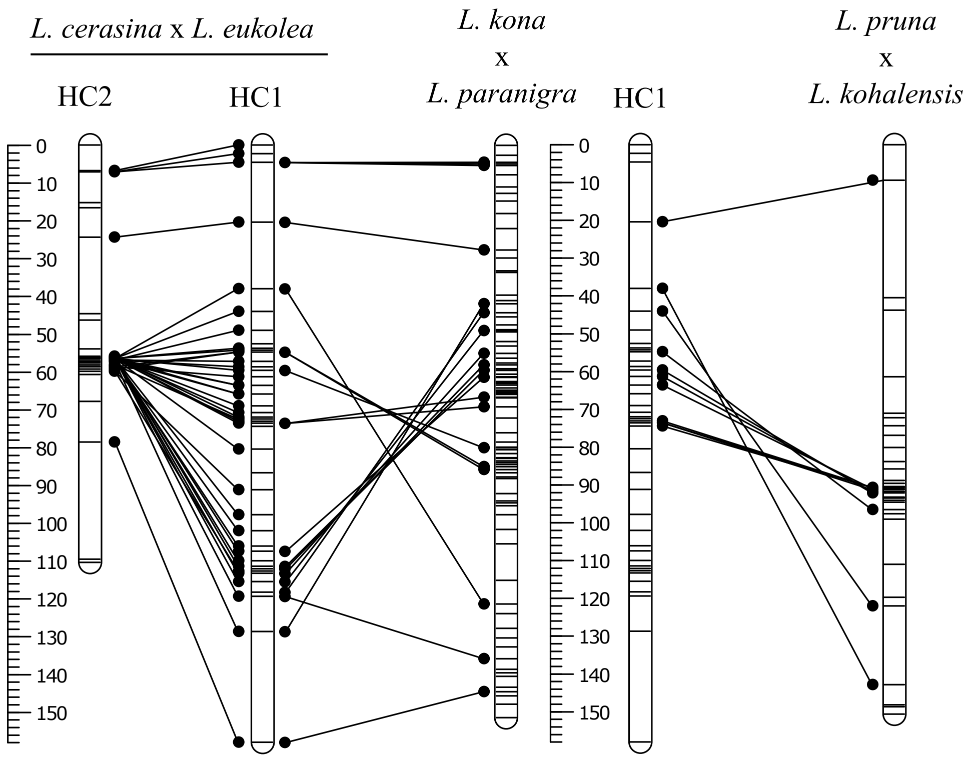

Figure S2. The region of extremely low recombination in HC2 relative to HC1 appears to be due to crossing parental lines with alternatively oriented inversion karyotypes. Comparing the marker order in HC1 with the marker order on homologous linkage groups in two other interspecies linkage maps (see Blankers et al. 2018 for details), shows that the marker order in HC1 is inverted relative to the other maps. Although further investigation is needed, the pattern suggests that a large pericentric inversion is segregating in either *L. cerasina* or *L. eukolea*, or both.
