# Supplementary material for: The Genetics of a Behavioral Speciation Phenotype in an Island System
Source: Genes (Basel). 2018 Jul 10;9(7):346. doi: 10.3390/genes9070346 (PMC6070818; doi:10.3390/genes9070346)
Supplement: Supplementary file 1 [file genes-09-00346-s001.zip › FigS1.pdf]

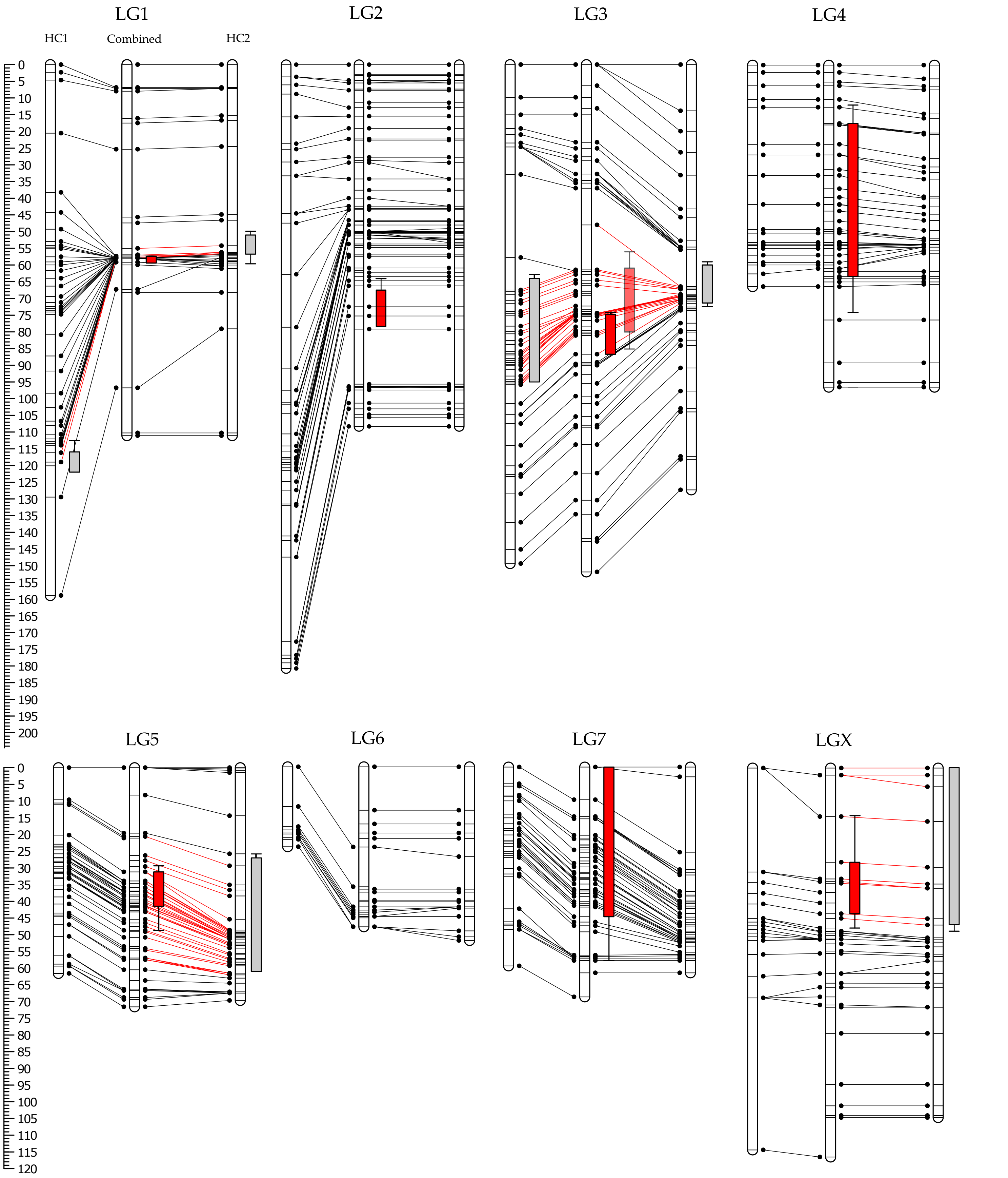


Figure S1. Linkage map and multivariate QTL model results. The horizontal black bars show the position of the SNP markers in the HC1, combined, and HC2 map, respectively. The black dots and solid lines connect markers on scaffolds shared between adjacent linkage groups. The scale indicates the position along the linkage groups in cM. The grey/red vertical rectangles and error bars show the 1-LOD and 1.5-LOD interval around the QTL peaks, respectively. We adjusted the 1.5-LOD interval for the HC1 QTL on LG1 to correct for the "phantom" peak at 37 cM. Red lines connect shared scaffolds within the 1-LOD intervals. On LG3, the partially transparant LOD interval indicates the position of the LOD interval in the model when only considering QTL on LG1, 3, 5, and X.
